# Surface components and metabolites of probiotics for regulation of intestinal epithelial barrier

**DOI:** 10.1186/s12934-020-1289-4

**Published:** 2020-02-05

**Authors:** Qing Liu, Zhiming Yu, Fengwei Tian, Jianxin Zhao, Hao Zhang, Qixiao Zhai, Wei Chen

**Affiliations:** 1 State Key Laboratory of Food Science and Technology, Jiangnan University, Wuxi, 214122 Jiangsu People’s Republic of China; 2 School of Food Science and Technology, Jiangnan University, Wuxi, 214122 Jiangsu China; 3 Wuxi People’s Hospital Affiliated to Nanjing Medical University, Wuxi, 214023 Jiangsu People’s Republic of China; 4 National Engineering Research Center for Functional Food, Jiangnan University, Wuxi, 214122 Jiangsu China; 5 (Yangzhou) Institute of Food Biotechnology, Jiangnan University, Yangzhou, 225004 China; 6 International Joint Research Laboratory for Probiotics at Jiangnan University, Wuxi, 214122 Jiangsu China; 7 Wuxi Translational Medicine Research Center and Jiangsu Translational Medicine Research Institute Wuxi Branch, Wuxi, China; 8 Beijing Innovation Centre of Food Nutrition and Human Health, Beijing Technology and Business University (BTBU), Beijing, 100048 People’s Republic of China

**Keywords:** Probiotic, Microbial-associated molecular patterns, Metabolites, Intestinal epithelial barrier

## Abstract

The gut microbiota can significantly affect the function of the intestinal barrier. Some intestinal probiotics (such as *Lactobacillus*, *Bifidobacteria*, a few *Escherichia coli* strains, and a new generation of probiotics including *Bacteroides thetaiotaomicron* and *Akkermansia muciniphila*) can maintain intestinal epithelial homeostasis and promote health. This review first summarizes probiotics’ regulation of the intestinal epithelium via their surface compounds. Surface layer proteins, flagella, pili and capsular polysaccharides constitute microbial-associated molecular patterns and specifically bind to pattern recognition receptors, which can regulate signaling pathways to produce cytokines or inhibit apoptosis, thereby attenuating inflammation and enhancing the function of the gut epithelium. The review also explains the effects of metabolites (such as secreted proteins, organic acids, indole, extracellular vesicles and bacteriocins) of probiotics on host receptors and the mechanisms by which these metabolites regulate gut epithelial barrier function. Previous reviews summarized the role of the surface macromolecules or metabolites of gut microbes (including both probiotics and pathogens) in human health. However, these reviews were mostly focused on the interactions between these substances and the intestinal mucosal immune system. In the current review, we only focused on probiotics and discussed the molecular interaction between these bacteria and the gut epithelial barrier.
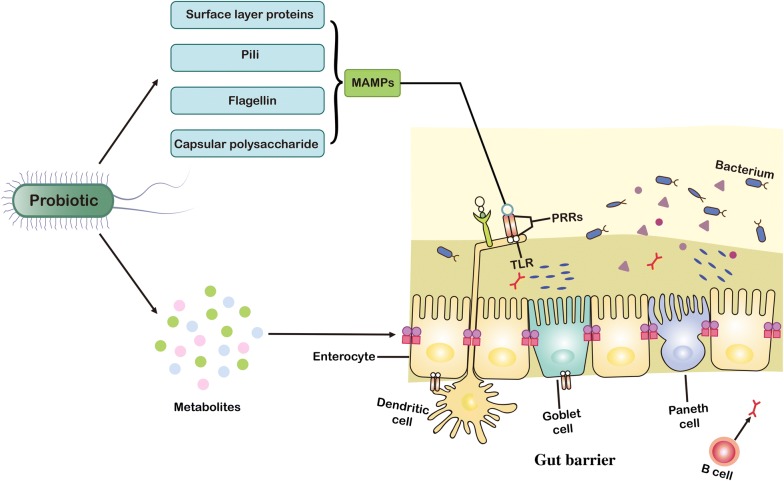

## Background

**Fig. 1:**
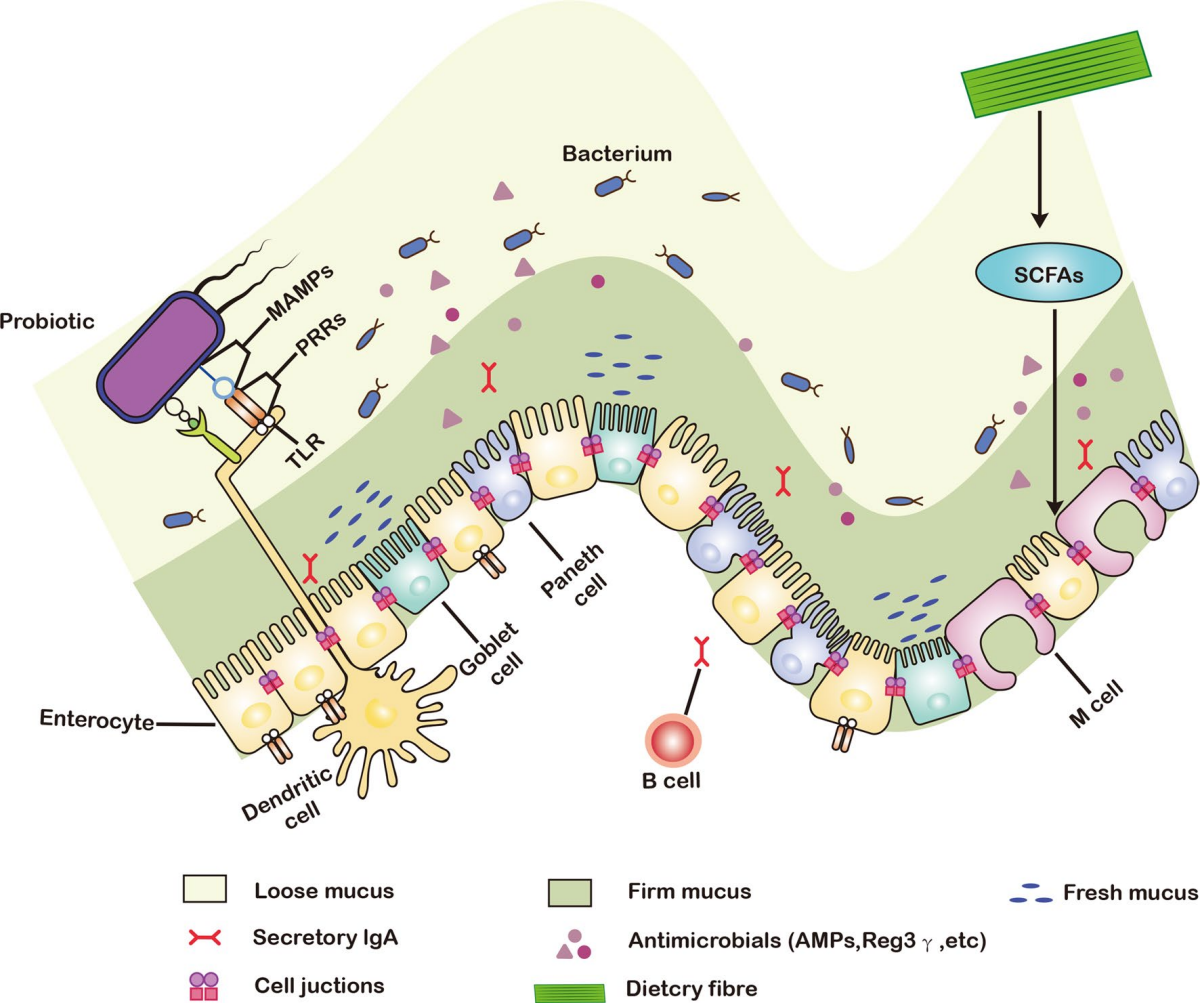
Structure, function, and probiotics of intestinal epithelial barrier. The mucus secreted by goblet cells continuously replenishes the mucosal layer that covers the intestinal epithelium, which acts as the first physical barrier against pathogenic bacteria. The symbiotic bacteria in the outer mucus layer can ferment dietary fiber into SCFAs, providing important energy sources for colonic intestinal cells and goblet cells. Paneth cells secrete a variety of antibacterial substances, such as antimicrobial peptides and Reg3γ. These antibacterial substances and secretory IgA are secreted into mucus to protect against commensal pathogens. The microorganism-associated molecular patterns (MAMPs) of probiotics can be recognized by PRRs such as TLRs, which induces the response of dendritic cells (DCs) to provide the protection on gut epithelial barrier. *PRRs* pattern recognition receptors, *SCFAs* short-chain fatty acids

The gut is a diversiform microenvironment in which hundreds of types of bacteria grow [1]. Intestinal epithelial cells (IECs) are generally considered to be immune sentinels and to play a crucial role in maintaining the integrity of the host’s intestinal mucosa [2]. Structurally, the monolayer of IECs separates the mucus produced by the goblet cells and the microbiota from the underlying immune cells to form a gut epithelial barrier (Fig. 1) [3]. The intestinal epithelial barrier is thus the main defense mechanism against infection and inflammation, and the disruption of its integrity is one of the primary causes of several intestinal disorders [4], including inflammatory bowel disease, necrotizing enterocolitis, diabetes, obesity, and irritable bowel syndrome [5]. Although gut diseases have a certain relationship with factors such as diet, genetics, and the environment, it is generally believed that dysbacteriosis is the most important factor that affects the intestinal barrier [6].

Probiotics are defined as “live microorganisms which when administered in adequate amounts confer a health benefit on the consumer” [7]. Commonly recognized intestinal probiotics include *Lactobacillus*, *Bifidobacterium*, *Streptococcus*, and a few *Escherichia coli* strains [1]. Recent studies have also indicated that some intestinal symbiotic bacteria such as *Akkermansia muciniphila* and *Bacteroides thetaiotaomicron* demonstrate the potency to comprise a new generation of probiotics [8, 9]. These bacteria have long been proven to regulate intestinal epithelial function by facilitating the formation of mucous layers, secreting antibacterial factors, boosting the secretion of secretory immunoglobulin A (SlgA) and competitive adhesion to intestinal epithelial cells [10, 11], and increasing tight junction formation [12]. Although these protective effects have been well documented, the underlying molecular mechanism of probiotics on the gut barrier has not been thoroughly reviewed.

The surface components of probiotics, such as flagella, pili, surface layer proteins (SLPs), capsular polysaccharide (CPS), lipoteichoic acid, and lipopolysaccharide, constitute microbial-associated molecular patterns (MAMPs) [13]. They can specifically bind to pattern recognition receptors (PRRs) such as NOD-like receptors (NLRs) and toll-like receptors (TLRs) (Table 1) [14, 15], and regulate nuclear factor kappa B (NF-κB), mitogen-activated protein kinases (MAPK), peroxisome proliferator-activated receptor gamma, and other signaling pathways in IEC [16]. MAMPs also regulate a cellular protease-dependent signaling cascade to produce a variety of cytokines and chemokines that alleviate inflammation and enhance intestinal epithelial function [10, 17]. In addition, some metabolites produced by probiotics, such as secreted proteins (extracellular proteins), organic acids, indole, bacteriocins, H_2_O_2_, and NO, protect the gut’s epithelial barrier by boosting mucus secretion by goblet cells, increasing the production of antimicrobial peptides, or enhancing the expression of tight junctions (Fig. 1) [18].

**Table 1:** Examples of interactions between MAMPs of probiotics and PRRs of hosts

MAMP	Probiotic				
	PRR	PRR location	Co-receptor	Species	Refs
SlpA	DC-SIGN	Cell membrane	Unknown	*L. acidophilus*	[29]
Flagellin	TLR5	Cell membrane	Unknown	*E. Coli* Nissle 1917	[35]
Pili	TLR4	Cell membrane	Mannose glycoproteins	*E. Coli* Nissle 1917 (type 1 pili)	[42]
CPS	Unknown	Unknown	Unknown	*B. thetaiotaomicron*	[48]
LTA	TLR2	Cell membrane	CD14 and CD36	*L. plantarum*	[113]
PG	TLR2-NOD1 (or NOD2)	Cell membrane–Cytoplasmic	CD14	*L. plantarum* DAP-PG	[114]
p40 p75	Unknown	Unknown	EGFR	*L. rhamnosus* GG	[55]
Indole	TLP4	Cell membrane	Unknown	*B. infantis*	[65]

*PRRs* pattern recognition receptors, *MAMPs* microbial-associated molecular patterns, *TLRs* toll-like receptors, *EGFR* epidermal growth factor receptor, *DC-SIGN* dendritic cell specific intercellular adhesion molecule grabbing nonintegrin, *Slp* surface layer protein, *CPS* capsule polysaccharide, *NOD* nucleotide binding oligomerization domain containing protein, *LPS* lipopolysaccharide, *LTA* lipoteichoic acid; p75 and p40, cell wall associated hydrolase, *PG* peptidoglycan

Based on the above mentioned analyses on the potential role of the surface compounds and metabolites of probiotics in gut barrier function, [10–13, 18] this review provides updated and comprehensive information on the molecular interaction between intestinal probiotics and the gut barrier and summarizes the effects of the surface macromolecules and metabolites of probiotics on intestinal receptors and pathways.

## Regulation of intestinal barrier function by surface molecules of probiotics

**Fig. 2:**
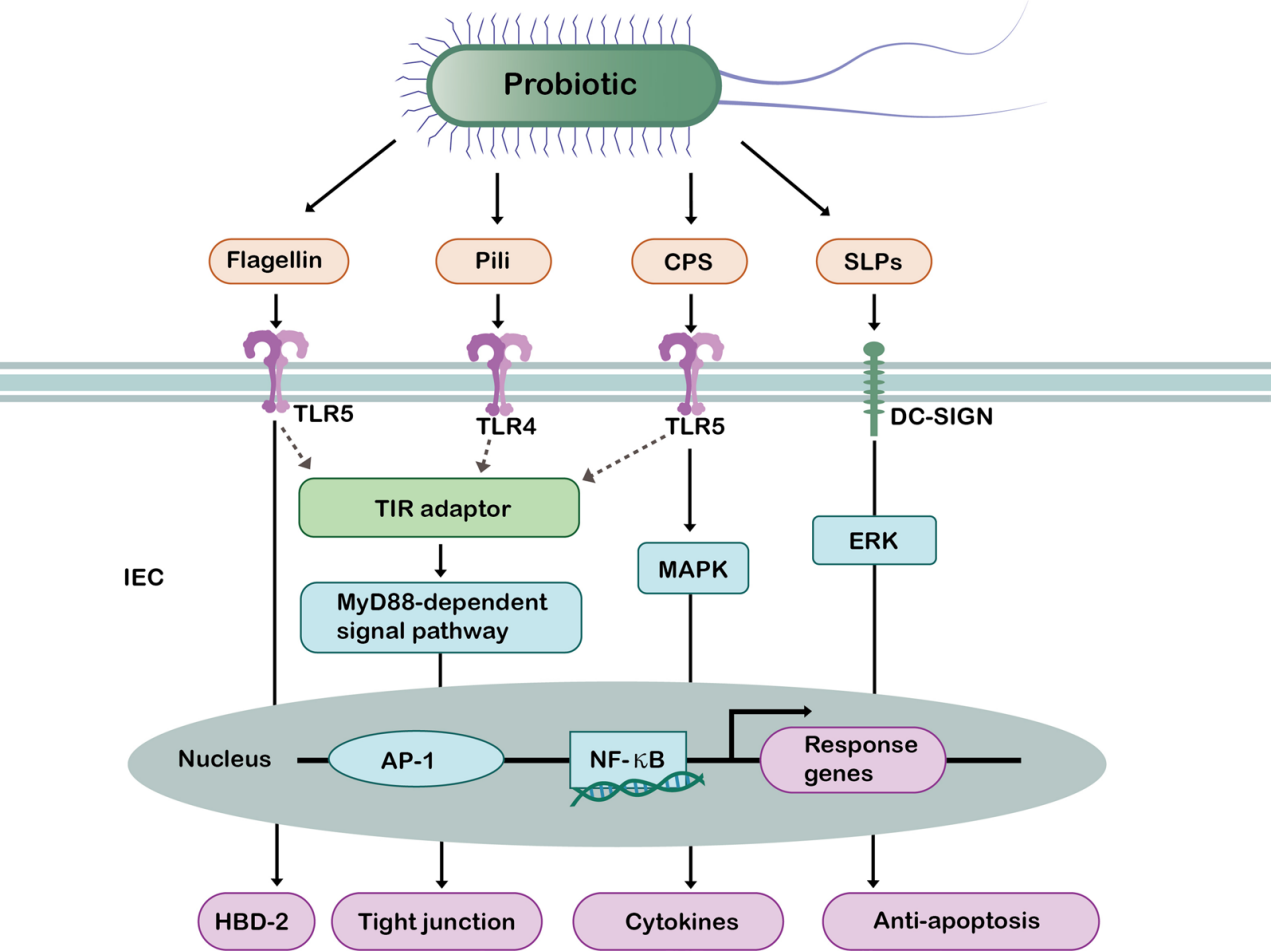
Effects of surface molecular of probiotics on intestinal epithelial barrier. Flagellin, pili, and CPS can be bind to TIR domain in TLRs, thus interacting with adaptor molecules such as MyD88 to activate AP-1 and NF-κB signaling pathways in IEC. Flagellin of EcN can finally induce the expression of HBD-2 in the gut, which is beneficial for the prevention of pathogens. F1C pili of EcN can finally up-regulate the expression of tight junction to enhance gut barrier function. CPS of EcN can finally induce the secretion of cytokines such as IL-10 and IL-12 for the alleviation of intestinal inflammation. SlpA of *Lactobacillus acidophilus* can bind to DC-SIGN and increase ERK phosphorylation, which mediates interaction with NF-κB and then reduce the expression level of cell apoptosis. *SLPs* surface layer proteins, *CPS* capsular polysaccharide, *TLRs* toll-like receptors, *DC-SIGN* dendritic cell specific intercellular adhesion molecule grabbing nonintegrin, *NF-κB* nuclear factor kappa B, *AP-1* activating protein-1, *IECs* intestinal epithelial cells, *ERK* extracellular signal-regulated kinase, *MAPK* mitogen-activated protein kinase, *HBD-2* beta-defensin 2

A number of previous studies have shown that the surface molecules of probiotics including SLPs, flagella, fimbriae and CPS can be recognized by PRRs and play a role in maintaining intestinal homeostasis and promoting gut health (Fig. 2) [13, 14, 16].

## Surface layer proteins

Bacterial surface layers are supramolecular cell envelope structures that are abundant in *Archaea* and in Gram-negative and Gram-positive bacteria [19, 20]. Chemical analyses of isolated S-layers showed that they are mostly composed of a single species of protein or multiple species of glycoproteins, with apparent relative molecular weights of 40,000 to 200,000 [21, 22]. These proteins were named as S-layer proteins (SLPs) [21, 22]. SLPs form a regular lattice monolayer via self-assembly and attach to the extracellular membrane by noncovalent interactions [21, 23]. As the outermost structure of the cell, the surface layer lattice is generally considered to be the first bacterial components that have a direct interaction with the host’s epithelium.

In previous studies, *L. helveticus* R0052 inhibited the adhesion of *E. coli* O157:H7 to Caco-2 cells [24], and its surface protein extract was able to co-aggregate with *Salmonella typhimurium* FP1 [25]. The function of SLPs in bacterial adhesion and gut barrier protection can be attributed to SLPs’ competition with pathogens such as enterohemorrhagic *E. coli* (EHEC), enteroinvasive *E. coli* (EIEC) and enteropathogenic *E. coli* (EPEC) for adhesion sites on the intestinal cell surface. It can also be attributed to their surface hydrophobicity [26], surface charge distribution [27], and co-aggregation of pathogenic bacteria [19].

A recent study indicated that purified SLPs from *L. plantarum* exert a protective effect on Caco-2 cells infected with EPEC by increasing their transepithelial resistance (TER) and down-regulating their permeability [28]. The SLPs of *L. acidophilus* have also been reported to protect the intestinal epithelium and inhibit its invasion by *Salmonella enterica* serovar Typhimurium by recovering TER [29]. SLPs can protect the intestinal barrier by affecting F-actin distribution and modulating the tight junction proteins at the mRNA and protein levels [30]. They can also increase extracellular signal-regulated kinase (ERK) phosphorylation, reducing the level of cell apoptosis [28].

Micro integral membrane proteins (MIMPs) were identified as the smallest domain from the SLPs of *L. plantarum* [31]. Previous studies have shown that MIMPs of *L. plantarum* CGMCC 1258 can restore tight junctional injury by increasing the expression of tight junction proteins including JAM-1, occludin, and claudin-1, which can allow the transportation of ions and small molecules of soluble substances through gut barrier, but prevent the passage of toxic large molecules and microorganisms [32].

## Flagellin

Flagellin is a structural component of bacterial flagella produced by pathogenic, symbiotic bacteria and neutral bacteria [33]. The interaction between flagellin and intestinal epithelium has mostly been studied on *E. coli* Nissle 1917 (EcN) [34]. Flagellin can induce inflammation in intestinal epithelial cells, whereas this proinflammatory effect is dismissed without contact with the basolateral membrane of the gut epithelia. This explains why flagellin-producing symbiotic microbes have not been found to induce inflammation in the gut lumen [35]. It has been reported that flagellin serves to activate phosphatidylinositol-3-kinase (PI3K)/AKT signaling pathway in the gut epithelium via a TLR5-dependent mechanism [36, 37]. The rapid activation of the PI3K pathway by TLR5 can limit the MAPK signaling pathway, thereby limiting the expression of proinflammatory genes and inhibiting inflammation [37]. It has also been reported that flagellin produced by the EcN can induce the secretion of beta-defensin 2 (HBD-2) [38], an antimicrobial peptide synthesized by intestinal epithelial cells. A follow-up study showed that the flagella-induced induction of HBD-2 is related to the NF-κB and activating protein-1 (AP-1) signaling pathways and thus offers antagonism against pathogens [34, 39]. It has been reported that the flagellum of the EcN, a main adhesin of intestinal mucous, can bind to receptors such as the mucus component gluconate and mediate its adhesion to mucin 2 [40]. These action modes can exclude pathogens and protect the intestinal epithelial barrier.

## Pili

Pili is a filamentous accessory organ on the surface of bacteria, which plays an important role in the adhesion between bacteria and host’s intestinal epithelium [41]. Pili is divided into 6 types (type I–type VI), based on their morphology, number, distribution on the surface of bacteria, adhesion characteristics, antigenicity and genetic locus [41]. Studies have revealed that EcN produces three main kinds of adhesins: F17-like pili, type 1 pili, and F1C pili [42]. Both F17-like and type 1 pili contribute to intestinal colonization and show significant binding to the epithelium in mice [42]. F1C pili can attach to mannosylated glycoproteins in the intestine and motivate TLR4 in a MyD88-dependent manner, thus improving the colonization and biofilm formation of EcN in the gut [42].

In vitro and in vivo experiments have demonstrated that the tight adhesion (Tad) pili of *B. breve* UCC2003 is a subclass of the type IVb pili. Tad has been reported to promote the proliferation of intestinal epithelial cells in mice [43]. The probiotic effect of *Bifidobacterium* Tad pili on the intestinal epithelial barrier can stimulate neonatal mucosal growth and intestinal maturation by producing a specific extracellular protein structural scaffold [44]. Subsequent reports have revealed that this beneficial proliferation response depends largely on the pili subunit TadE [44]. It has also been shown that SpaC fimbriae of probiotics are essential for adhesion to Caco-2 intestinal epithelium lines [45, 46]. The SpaC pilin of *L. rhamnosus* GG (LGG) has been confirmed to induce the generation of reactive oxygen species (ROS) in epithelium and play a role in stimulating ERK phosphorylation and protecting the gut’s epithelial barrier [47].

## Capsular polysaccharide

The CPS of bacteria is homopolymers or heteropolymers formed by repeated monosaccharides linked by glycosidic bonds [19]. CPS molecules in probiotics have a positive effect on adaptation to the intestinal microenvironment. *B. thetaiotaomicron* can express and dynamically transform various types of CPS in vivo, the most prevalent being CPS5, which can enhance the competition and colonization of bacteria in the gut of mice [48]. CPS5 also enhances the tolerance of *B. thetaiotaomicron* to antibiotic stress [48]. Furthermore, some studies revealed that the K5 capsule of EcN stimulates TLR5 in gut epithelial cells and induces chemokine expression via the mitogen-activated protein kinase pathway [49, 50].

To summarize, the surface substances of probiotics share a common regulatory mechanism as they can bind to PRRs including TLRs, NLRs, DC-SIGN and CLRs. Upon exposure to these surface substances, PRRs respond by activating associated adaptor proteins that are linked to NF-κB and MAPK signaling cascades, which further affects the expression of genes encoding cytokines, chemokines and antimicrobial peptides.

## Regulation of intestinal barrier function by main metabolites of probiotics

Some metabolites produced by probiotics, such as secreted proteins (extracellular proteins), indole, extracellular vesicles, short-chain fatty acids, and bacteriocins also protect the intestinal epithelial barrier by interacting with some receptors or directly promoting mucus secretion by goblet cells, increasing the secretion of antimicrobial peptides, or enhancing the expression of tight junctions [18].

## Secreted protein of probiotics

**Fig. 3:**
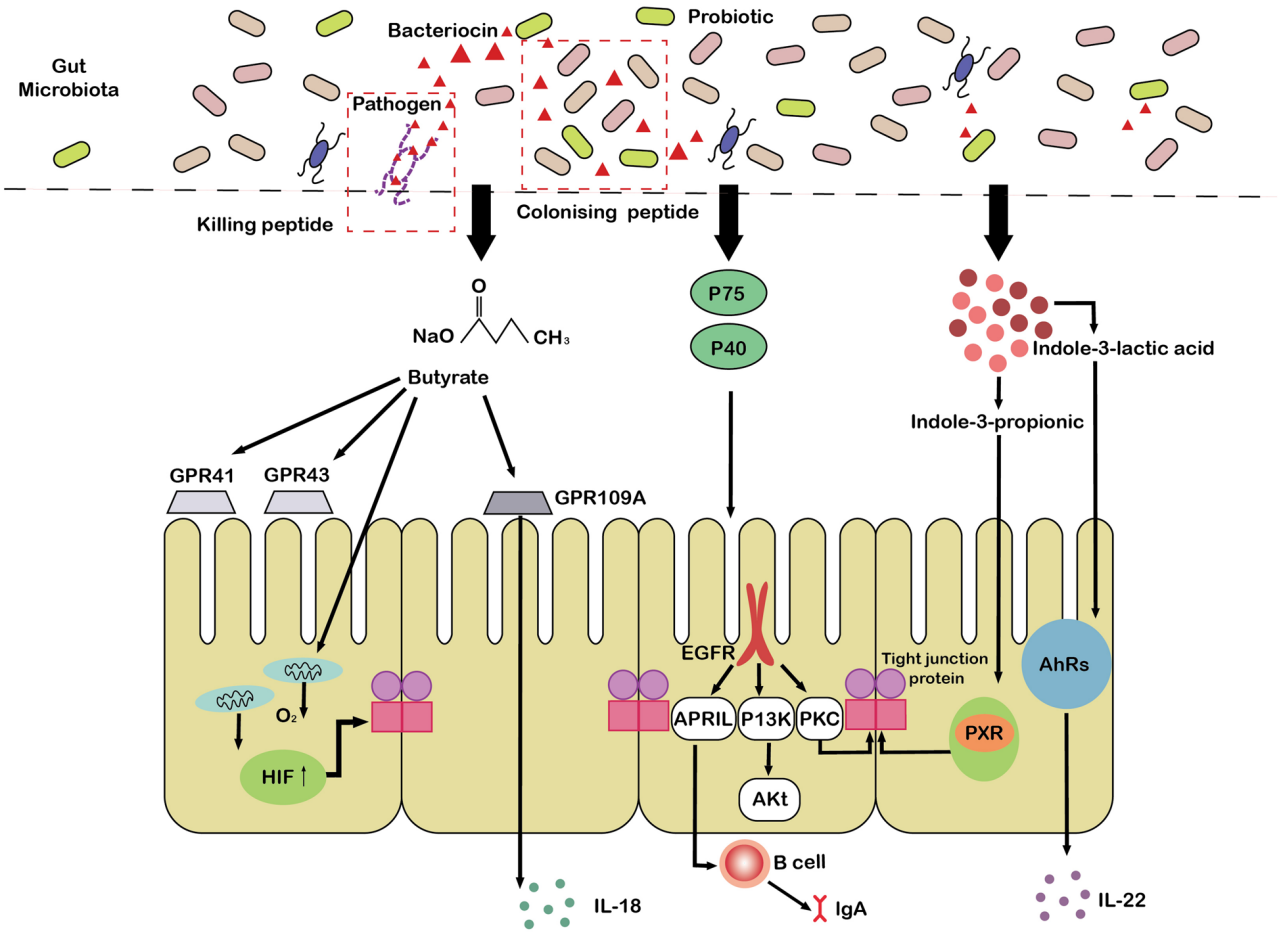
Effects of metabolites of probiotics on intestinal epithelial barrier. Indole 3-propionic acid can bind to PXR and up-regulate the expression of tight junction protein. The indole-3-lactic acid activates AhRs of the gut epithelium and promotes the expression of IL-22. The soluble proteins P40 and p75 isolated from LGG can activate EGFR and then up-regulate the expression of an APRIL in the epithelium, thus stimulating the secretion of lgA by B cells. Besides, P40 and p75 can activate EGFR–PIK3–Akt signaling pathway to maintain gut homeostasis. Moreover, these two proteins also prevent tight junctional disruption by protein kinase C (PKC)-dependent mechanisms. Butyrate is able to bind to the GPCR including GPR41, GPR109A, and GPR43 and induce the production of IL-18 in the colonic epithelium. Furthermore, butyrate also motivates the O_2_ consumption of the gut epithelium to maintain HIF stability and increase the expression of barrier-protective HIF target genes. In addition, bacteriocins produced by probiotics act as colonizing peptides to encourage producers to gain a competitive advantage over other strains and to occupy established niches in the intestines. Alternatively, bacteriocins can act as a killing peptide, directly inhibiting the adhesion of pathogens to the mucus layer and protecting the first barrier of the intestinal tract. *HIF* hypoxia-inducible factor, *GPR109A* G-protein-coupled receptors 109A, *AhRs* aryl hydrogen receptors, *P75 and P40* cell wall-associated hydrolase, *EGFR* epidermal growth factor receptor, *PI3K* phosphatidylinositol-3-kinase, *PKC* protein kinase C, *PXR* pregnane X receptor, *APRIL* a proliferation-inducing ligand, *PKC* protein kinase C

A number of previous studies indicated that secreted proteins (extracellular proteins) are proteins secreted and released into the environment by probiotic [51–53]. The secreted proteins of probiotics have also been reported to participate in the interaction between symbiotic bacteria and the host. The extracellular proteins secreted by *L. plantarum* BMCM12 effectively attenuate the adherence of pathogens and protect the intestinal barrier [51]. Two proteins produced by LGG, p40 and p75, have been shown to promote IEC homeostasis. The mechanism is as follows. First, the soluble proteins P75 and p40 transactivate the epidermal growth factor receptor (EGFR) [52] and then up-regulate the expression of a proliferation-inducing ligand (APRIL) in the epithelium (Fig. 3) [53]. This in turn promotes the production of immunoglobulin A and attenuates cytokine-induced apoptosis in mouse small intestine epithelial cells [53]. Second, these two proteins stimulate the intestinal epithelial cells to produce protective heat stress proteins Hsp72 and Hsp25, which protect tight junction proteins and activate the Akt pathway in a phosphatidylinositol 3-kinase (PIK3)-dependent manner to enhance the proliferation and survival of gut epithelial cells (Fig. 2) [54]. Alternatively, other studies have demonstrated that neonatal supplementation of P40 and p75 can promotes intestinal development and prevents colitis in adulthood [55, 56]. Moreover, these two proteins also prevent H_2_O_2_-induced tight junctional disruption by protein kinase C (PKC)-dependent mechanisms [57].

Similarly, a novel LGG-soluble protein HM0539, has been reported to protect intestinal integrity by mediating tight junction expression and mucus secretion [58]. Furthermore, Ewaschuk et al. used a mouse model with and without interleukin (IL)-10 and found that an extracellular protein secreted by *B. infantis* positively regulated occludin and ZO-1 proteins and increased TER, thus reducing colonic permeability and strengthening the mucosal barrier [59].

## Indole

Indole is usually produced by bacteria that contain tryptophanase and has been reported to be a specific intestinal symbiotic bacteria signal [60, 61]. Studies have indicated that indole produced by symbiotic *E*. coli can inhibit the chemotaxis of pathogenic *E. coli* [62]. *E. coli*-secreted indole can also inhibit the attachment of pathogens to the epithelium by increasing the expression of genes involved in intestinal epithelial function, such as actin cytoskeleton, adhesion junctions, and tight junctions [63]. Furthermore, this bacterial signal increased TER in polarized HCT-8 gut epithelium and attenuated tumor necrosis factor α-mediated NF-κB activation and IL-8 secretion, thus facilitating epithelial function [63].

The pregnane X receptor (PXR) is a physiologic regulator associated with gut permeability, which is considered to regulate the intestinal barrier mediated by TLR4 [64–66]. Indole 3-propionic acid (IPA) has been reported as a ligand for epithelial PXR [61, 67], and the administration of IPA can up-regulate tight junction protein-coding mRNAs and augment the expression of claudins and occludins [65]. It has been reported that the indole-3-lactic acid produced by *B. infantis* activates the aryl hydrogen receptors (AhRs) of the gut epithelium by increasing their nuclear localization and up-regulating the protein expression of CYP1A1 [68]. The activation of AhRs then leads to lL-22 transcription, which can further increase the expression of antimicrobial peptides and improve colonization resistance against *Candida albicans* in the gastrointestinal tract [68].

## Extracellular vesicles

Extracellular vesicles (EVs), nanoscale membrane vesicles, are lipid bilayer structures secreted by the intestinal microbiota that are composed mainly of nucleic acids, proteins, lipids, and lipopolysaccharides [69, 70]. EVs are involved in bacteria-host communication and in the maintenance of gut homeostasis. It has been reported that oral application of A. muciniphila derived EVs can alleviate dextran sulfate sodium-induced colitis by recovering inflammatory cell infiltration of the colon wall and alterations in colon length [71]. These phenomena can be explained by the fact that *A. muciniphila* derived EVs up-regulate the expression of claudin-3 and reduce intestinal permeability in diabetic mice in an AMP-activated protein kinase (AMPK)-dependent manner [72–74].

The EVs of most bacteria are obtained by blistering the outer membrane and ultimately pinching off the bacterial cytoderm, so they are referred to as outer membrane vesicles (OMVs). Studies have shown that OMVs secreted by *E. coli* ECOR63 and EcN can upregulate tight junction proteins such as claudin-14 and ZO-1 [75, 76]. Probiotic EcN derived OMVs can also induce IL-22 expression in colonic explants, thereby preventing allergens and pathogenic microorganisms from entering the systemic circulation [75].

## Short-chain fatty acids

Short-chain fatty acids, which comprise mainly butyrate, propionate, and acetate, are metabolites secreted by intestinal microbiota from undigested dietary carbohydrates and proteins [77]. As butyrate is the preferential source of energy for colonic epithelial cell among all short-chain fatty acids, the relationship between butyrate and the intestinal epithelial barrier is the most-studied [78].

Studies have revealed the protective effect of a low concentration of butyrate (≤ 2 mM) on the single-layer barrier of Caco-2 cells, such as the increase in TER and the decrease in inulin permeability [79, 80]. Moreover, microbial-derived butyrate boosts the expression of tight junction proteins and represses paracellular permeability in vivo [81], and it stimulates goblet cells to secrete mucin, especially MUC2, which prevents pathogenic bacteria from destroying enterocytes [82]. A mucin-related peptide that can repair the intestinal mucosa, trefoil factor, can also be upregulated by butyrate [77]. Butyrate contributes to activate hypoxia-inducible factor (HIF) in the hypoxic region of the colon, which further promotes intestinal epithelial barrier function, antimicrobial defense, and mucus production [83, 84].

Butyrate is a histone deacetylase inhibitor and has been reported to bind to specific G-protein-coupled receptors, including GPR109A, GPR43, and GPR41 [85, 86]. Of these, GPR109A is crucial for the production of IL-18 in the colonic epithelium and has been confirmed to have an important effect on the maintenance of intestinal homeostasis (Fig. 3) [81, 87]. One of the mechanisms by which butyrate improves gut epithelial barrier function is the activation of AMP-activated protein kinase [87, 88]. Second, low concentrations of butyrate can augment the MUC2 mRNA level by promoting AP-1 binding to the MUC2 promoter [82]. At the same time, butyrate can boost the acetylation of histones H4 and H3 and the methylation of H3 on the MUC2 promoter, thereby safeguarding the mucosal barrier [82]. Butyrate also inhibits permeability-promoted claudin-2 tight junction protein expression via an IL-10RA-dependent mechanism [89]. Furthermore, the production of antimicrobial cathelicidin, such as LL-37 in the body has also been specifically linked to butyrate [90]. In addition, butyrate can motivate the O_2_ consumption of the gut epithelium to the extent of HIF stability and increase the expression of barrier-protective HIF target genes, connecting microbes and epithelial barriers (Fig. 3) [91, 92].

## Bacteriocins

Bacteriocins are a class of ribosomally synthesized antimicrobial peptides [93–95] and can be divided into two specific classes: lanthionine-containing bacteriocins/lbacteria (class I) and non-lanthionine-containing bacteriocins (class II). [96]. The class I bacteriocins comprise single peptide chain and polypeptide chain lantibiotics. These bacteriocins, including lacticin 481, lacticin 3147, and nisin, are ribosomally-synthesised antimicrobial peptides produced by Gram-positive bacteria. [97, 98]. The class II bacteriocins are mainly composed of subclass I, subclass II, subclass III and subclass IV. The common bacteriocins in class II are pediocin pa-1, lactacin F, lactococcin A and reuterin 6. We have added an introduction to the classification of bacteriocins [99].

Bacteriocins have been reported to act as colonizing peptides of certain intestinal micro-organisms, promoting these bacteria to acquire a competitive advantage over other strains and occupy established niches in the intestines [100]. Studies have shown that EcN can secrete microcin H47 and microcin M, two antimicrobial peptides with low molecular weight that can be discerned by the catecholate siderophore receptors and thus enhance the competitiveness of EcN with other microorganisms [101]. Bacteriocin produced by the strain *Enterococcus faecium* KH24 conspicuously affects the microbiome in the feces of mice [102]. In addition to reducing the number of *E. coli*, this bacteriocin can significantly increase the abundance of *Lactobacillus* [102].

Alternatively, bacteriocins function as killing peptides since they can interfere with the growth of pathogens (especially Gram-negative bacteria) by penetrating the inner membrane or disrupting cell wall synthesis. [103]. *L. reuteri* can secrete a secondary metabolite with broad-spectrum antibacterial activity, called reuterin, that directly inhibits pathogens [104]. Moreover, nisin, which is mainly produced by *Streptococcus lactis* and *Lactococcus lactis*, can restrain the growth and reproduction of most Gram-positive bacteria and their spores, especially against *S. aureus* and *Streptococcus hemolyticus* [105]. Furthermore, the class II bacteriocin Abp118 secreted by *L. salivarius* UCC118 can prominently protect mice from infection by *Listeria monocytogenes* [106]. In addition, EntV produced by *E. faecalis* bacteria represses hyphae and biofilm formation in *Candida albicans* and reduce the virulence of this fungus [107].

## Conclusions

Probiotics and gut commensals can modulate the host’s gut epithelial barrier function via their surface molecules and metabolites. Through organoid models, sterile animal models, and in vitro tissue, we may better characterize the impact of the intestinal microflora on the host epithelium. Surface components and metabolites of probiotics can be further used in clinical studies and dietary interventions for the treatment of diseases associated with specific intestinal barriers [108–112].

## Data Availability

Not applicable.

## Abbreviations

MAMPs: Microbial-associated molecular patterns
PRRs: Pattern recognition receptors
NLRs: NOD-like receptors
TLRs: Toll-like receptors
NF-κB: Nuclear factor kappa B
MAPK: Mitogen-activated protein kinases
SlgA: Secretory immunoglobulin A
SLPs: Surface layer proteins
TER: Transepithelial resistance
ERK: Extracellular signal-regulated kinase
PI3K: Phosphatidylinositol-3-kinase
HBD-2: Beta-defensin 2
EcN: *Escherichia coli* Nissle 1917
Tad: Tight adhesion
CPS: Capsular polysaccharide
ROS: Reactive oxygen species
EGFR: Epidermal growth factor receptor
APRIL: A proliferation-inducing ligand
PXR: The pregnane X receptor
IPA: Indole 3-propionic acid
AhRs: Aryl hydrogen receptors
EVs: Extracellular vesicles
OMVs: Outer membrane vesicles
HIF: Hypoxia-inducible factor
PKC: Protein kinase C
Dgk: Diacylglycerol kinase
